# The Molecular Mechanism of the Response of Rice to Arsenic Stress and Effective Strategies to Reduce the Accumulation of Arsenic in Grain

**DOI:** 10.3390/ijms25052861

**Published:** 2024-03-01

**Authors:** Anjing Geng, Wenli Lian, Yihan Wang, Minghao Liu, Yue Zhang, Xu Wang, Guang Chen

**Affiliations:** 1 Institute of Quality Standard and Monitoring Technology for Agro-Products of Guangdong Academy of Agricultural Sciences, Guangzhou 510640, China; 2 Key Laboratory of Testing and Evaluation for Agro-Product Safety and Quality, Ministry of Agriculture and Rural Affairs, Guangzhou 510640, China; 3 Guangdong Provincial Key Laboratory of Quality & Safety Risk Assessment for Agro-Products, Guangzhou 510640, China

**Keywords:** rice, arsenic, molecular mechanism, agronomic practices, biomolecular technology

## Abstract

Rice (*Oryza sativa* L.) is the staple food for more than 50% of the world’s population. Owing to its growth characteristics, rice has more than 10-fold the ability to enrich the carcinogen arsenic (As) than other crops, which seriously affects world food security. The consumption of rice is one of the primary ways for humans to intake As, and it endangers human health. Effective measures to control As pollution need to be studied and promoted. Currently, there have been many studies on reducing the accumulation of As in rice. They are generally divided into agronomic practices and biotechnological approaches, but simultaneously, the problem of using the same measures to obtain the opposite results may be due to the different species of As or soil environments. There is a lack of systematic discussion on measures to reduce As in rice based on its mechanism of action. Therefore, an in-depth understanding of the molecular mechanism of the accumulation of As in rice could result in accurate measures to reduce the content of As based on local conditions. Different species of As have different toxicity and metabolic pathways. This review comprehensively summarizes and reviews the molecular mechanisms of toxicity, absorption, transport and redistribution of different species of As in rice in recent years, and the agronomic measures to effectively reduce the accumulation of As in rice and the genetic resources that can be used to breed for rice that only accumulates low levels of As. The goal of this review is to provide theoretical support for the prevention and control of As pollution in rice, facilitate the creation of new types of germplasm aiming to develop without arsenic accumulation or within an acceptable limit to prevent the health consequences associated with heavy metal As as described here.

## 1. Introduction

Arsenic (As) is a naturally occurring metalloid that is found in terrestrial and aquatic environments in more than 105 countries and regions [1]. As has four valence states, including −3, 0, +3 and +5. It is primarily divided into inorganic As—such as arsenite [As(III)] and its salts and arsenate [As(V)] and its salts—and organic As—such as monomethylarsenic (MMA), dimethylarsenic (DMA) and trimethylarsenic (TMA) [2]. The +3 and +5 valences are the most common in aqueous environments. The +3 valence is dominant under reducing conditions, while the +5 valence is dominant under oxidizing conditions [3,4].

Arsenic is classified as a class I carcinogen by the International Agency for Research on Cancer (IARC) [5]; the health hazards of As manifest in both carcinogenic and non-carcinogenic forms. The kinds of cancer that have been reported include skin cancer, lung cancer, bladder cancer, liver cancer, prostate cancer, and leukemia, and the non-carcinogenic effects include neurological effects, memory and intellectual function, diabetes, skin disorders, cardiovascular disorders, reproductive system disorders, etc. [6]. The direct carcinogenic mechanism of As is to generate multiple types of DNA damage induced by oxidative stress and reactive oxygen species (ROS), and ROS generation and DNA repair inhibition play important roles in the co-carcinogenicity mechanisms of As indirectly [7]. Approximately 220 million people worldwide may be at risk of drinking As-contaminated groundwater [8]. Soil is the primary way in which crops are contaminated with As. More than 105 countries have reported natural sources of As contamination in groundwater with As concentrations that range from 0.5 to 5000 μg L^−1^, while the World Health Organization (WHO) limits the concentrations of As in water to 10 μg L^−1^ [9]. So, As pollution is considered to be one of the most problematic pollutants.

Owing to its growth characteristics, rice (*Oryza sativa* L.) more easily absorbs As than other crops. Studies have shown that the amount of As that accumulates in rice is 10-fold higher than that of other food crops, such as wheat (*Triticum aestivum*) and barley (*Hordeum vulgare*) [10]. The long-term consumption of rice that contains As will lead to the accumulation of As in the human body [11], leading to many health hazards such as cancers. A study that covered a variety of potential sources of As in the human body showed that 56% of the total As in the human body is derived from rice [12]. Different species of As vary in their degree of toxicity. Typically, the most toxic compounds are shown as follows: As(III) > As(V) > MMA > DMA, and the toxicity of As(III) is 100-fold higher than that of As(V) [13]. Therefore, it is necessary to consider the species of As when studying the molecular mechanism of the response of rice to As toxicity. Studies have shown that 85–95% of the As in rice is inorganic [14]. Arsenic has no biological function in plants and exerts a negative impact on the growth, development, and yield of rice [15]. The yield of rice is also severely affected (about 90%) by a disease (straight head disease of rice) caused by DMA [16]. It is highly significant to understand the physiological and molecular mechanisms of the response of rice to As and propose effective measures to reduce the accumulation of As in rice, which is highly significant for solving the problem of As contamination in rice, improving the quality and safety of rice, and ensuring human health suffering from As toxicity. In this review, the important results of research related to the toxicity, absorption, transport, redistribution and metabolism of As in rice in recent years are summarized along with the agronomic measures and molecular strategies that can be used to reduce the accumulation of As in rice. The goal of this review is to provide a theoretical basis for the prevention and control of As in rice and the cultivation of new varieties that accumulate low levels of As.

## 2. Effects of As on the Growth, Development and Metabolism of Rice

**Figure 1:**
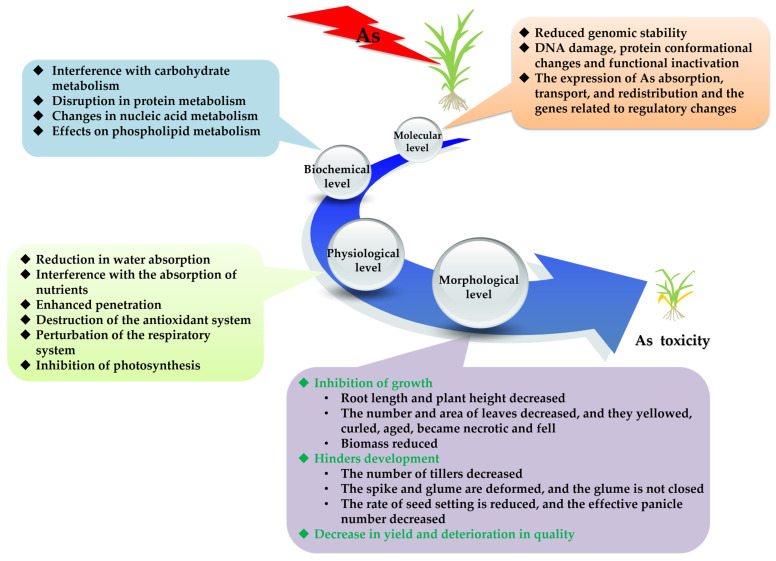
Effects of arsenic toxicity on rice.

Arsenic has a negative impact on rice at the morphological, physiological, biochemical and molecular levels. As stress produces excessive reactive oxygen species (ROS), reactive nitrogen species (RNS) and free radicals, among others, which destroy the structures of organelles, damage DNA and RNA and interfere with physiological and biochemical processes, such as carbohydrate, protein and nucleic acid metabolism, and thus, inhibit the growth and development of rice. This results in a decrease in biomass, rate of seed setting, yield and reduction in quality [17] (Figure 1).

When rice is stressed by As, it undergoes a series of significant changes at the molecular, physiological and biochemical levels, which, in turn, causes changes in the growth and development-related phenotypes and ultimately leads to a reduction in the yield and quality of rice and increased safety risks.

Arsenic affects the morphology and growth of rice. Stress owing to inorganic As results in a significant decrease in the rate of germination of the rice seeds, coleoptile length, root length, seed fresh weight, plant height, biomass and relative water content of the rice seedlings as the concentration of As increased [11,18]. In addition, the absorption and efficiency of the utilization of nitrogen (N), phosphorus (P) and potassium (K) in the rice seedlings tended to decrease, which affected its growth and yield [11]. DMA toxicity leads to a 90–100% reduction in the yield of rice [16]. When subjected to high levels of As treatment, the cell wall was degraded; the nucleus increased in size and lost its circular structure; more particles of metal aggregates appeared in the cytoplasm and organelles; the chloroplast was degraded; the spindle structure was lost; more cavities appeared inside; the grana and internal capsule were degraded; the arrangement was not tight; and photosynthesis was inhibited [18].

Arsenic affects the physiological metabolism of rice. As stress inhibited the activities of superoxide dismutase (SOD), peroxidase (POD) and catalase (CAT) in the rice roots and leaves, and the higher As concentration, the more inhibition there was [11], and the more the content of hydrogen peroxide (H_2_O_2_) and malondialdehyde (MDA) increased [19]. The photosynthetic rate, stomatal conductance and intercellular CO_2_ concentration was negatively correlated with the content of As in rice [20]. Under As stress, water transport was inhibited; proline was accumulated, and the rate of electrolyte leakage increased [21].

Arsenic affects the biochemical processes of rice. It can cause carbohydrate metabolism disorder, which leads to the accumulation of reducing, non-reducing and total soluble sugars by changing the activities of sucrase and other enzymes [22], while the content of protein decreases [23]. The activity of arsenate reductase in the roots of As-tolerant rice increased [24], and the essential amino acids and non-essential amino acids in the grains decreased with the increase in As concentration [25]. As binds to the thiol (-SH) and amino (-NH_2_) groups of proteins and changes their folding patterns and functions [26], thereby affecting the activities of these proteins.

Arsenic can also affect rice at the molecular level such as disruption of the stability of the genome and the expression of genes [27]. For example, Huang et al. (2019) found that 983 differentially expressed genes (DEGs) were up-regulated, and 520 DEGs were down-regulated in rice roots under As stress [28]. As stress inhibited the levels of expression of *OsNIP1;1*, *OsNIP2;1*, *OsABCG5*, *OsNRAMP1*, *OsNRAMP5*, and *OsTIP2;2* [29]. Exposure of the rice roots to 20 μM As(III) up-regulated the levels of expression of *OsPT19*, *OsACA1* and *OsALA4*, while the levels of expression of the genes that encoded citrate transporter and aquaporin decreased significantly. At a concentration of 80 μM As(III), the levels of expression of *OsNIP3;2*, *OsNIP1;1, OsHMA5* and *OsHMA9* in the roots were significantly up-regulated, while the levels of expression of *OsPT5*, *OsPT8*, *OsPT19* and *OsPT23* decreased [30].

The response of rice to As stress is affected by many factors, such as the variety, As species and concentration, and soil conditions. In particular, the toxicity of different species of As differs significantly. It is necessary to analyze the different species of As to evaluate the risk of As scientifically and reasonably.

## 3. Absorption, Transport and Redistribution of As(III) in Rice

### 3.1. Absorption of As(III)

As(III) is the dominant form and is absorbed primarily by rice in flooded paddy soil [31]. Since As(III) is an analog of boric acid and silicic acid [17], it is currently reported that As(III) is absorbed by the transmembrane transport of some subfamily members of the major intrinsic protein (MIP) family (Table 1), such as nodulin26-like intrinsic proteins (NIPs), plasma membrane intrinsic proteins (PIPs) and tonoplast intrinsic proteins (TIPs) [32]. Among these, the aquaporins NIPs are particularly important. The NIPs bidirectionally transport As(III), and the absorption and efflux of As(III) primarily depend on the difference in the concentration of As in the rice roots and growth media [33]. Many transporters can transport As(III), including OsNIP1;1 [34], OsNIP2;1 (OsLsi1) [35], OsNIP2;2 (OsLsi6), OsNIP3;1, OsNIP3;2 [36], OsNIP3;3 [34], OsPIP2;4, OsPIP2;6 and OsPIP2;7 [32,36]. Of these, OsNIP2;1 plays a leading role in the entry and exclusion of As(III) into the root cells. OsNIP3;2 is involved in the absorption of As(III) by the lateral roots [32,36]. OsNIP3;3 shows transport activity under As(III) stress [32]. In addition, small basic intrinsic proteins (SIP) and uncategorized intrinsic proteins (XIP) also regulate the absorption of As(III) through different modes of action [37]. Because As(III) is also similar to the structure of glycerol, it is easily absorbed by aquaglyceroporins [38]. It has also been reported that three As(OH)_3_ molecules can be polymerized to form a ring structure similar to that of hexose molecules, which is then absorbed by the hexose transporters [39]. There are three main routes that As(III) can take once it enters the rice roots: (1) Part of the As(III) is excreted from the root cells through OsLsi1. (2) It forms a complex with phytochelatin (PC), which is synthesized by phytochelatin synthase, and then transferred to the vacuoles by the transporter OsABCC1 [40]. (3) OsLsi2, OsNRAMP1 and other transporters transport As(III) to the xylem, and it is then transported upward to the stems, leaves and grains [37,41].

Currently, no specific transporter that mediates the absorption of As(III) has been identified, which makes it difficult to regulate the absorption of As(III) in rice by genetic engineering methods, such as silencing and knockout. It is necessary to further explore new genes that are specific for the absorption of As(III).

### 3.2. Transport of As(III)

As(III) can be transferred to the shoots through the silicon transporter OsLsi2 and accumulates in the grains. The loading and transport of As(III) to the xylem through OsLsi1 and OsLsi2 results in the accumulation of As(III) in the vegetative parts [60,61]. OsNRAMP1 promotes the xylem loading of As(III) [62]. In contrast, the transporters OsNIP1;1 and OsNIP3;3 on the plasma membrane of rice roots restrict the entry of As(III) into the xylem by expelling it from the stele [63]. The content of As in the nodes of rice was significantly higher than that in other parts of the shoots. The first node under the panicle was the filter for As(III) to enter the grain, which regulated the accumulation of inorganic As in the grains and leaves [41]. OsABCC1 isolates As in the associated vacuoles of rice nodes and plays an important role in limiting the transport of As(III) to the grains [42]. The transcription factor OsARM1 (ARSENITE-RESPONSIVE MYB1) regulates the absorption and transport of As(III) by inhibiting the levels of expression of *OsLsi1*, *OsLsi2* and *OsLsi6* [41,57]. OsGrx_C7 regulates the growth of roots and inhibits the transport of As(III) from the roots to shoots by reducing the levels of expression of *OsNIP1;1*, *OsNIP3;1*, *OsLsi1* and *OsLsi2* [58].

Fewer transporters have been reported to be involved in the long-distance transport of As(III) in rice than those that mediate the absorption of As(III). The function of different aquaporins in rice merits further study. In addition, the mechanism used by rice nodes to regulate the upward transport of As(III) merits further clarification.

### 3.3. Redistribution of As(III)

As(III) is primarily transported to the rice grains through the phloem [64]. OsLsi2 plays an important role in the accumulation of As(III) in the rice nodes and grains. The mutation of *OsLsi2* resulted in a significant decrease in the accumulation of As(III) in the phloem. When the in vitro panicles were exposed to As(III), more of this compound was distributed in the nodes and flag leaves of the *oslsi2* mutant compared with the wild type (WT), while less As(III) was distributed in the grains [41]. OsNIP6;1 and OsNIP7;1 also play an important role in the process by which As(III) enters the grains [65]. In addition, the OsABCC1 transporter inhibits the entry of As into rice by isolating As(III) in vacuoles [40]. In the presence of 10 µM As, the growth of both the roots and shoots of the knockout lines was inhibited to a greater extent than that of WT rice. The shoot growth of the knockout lines was completely inhibited in the presence of 50 µM As [40]. The content of As in the nodes of *osabcc1* knockout mutants was lower than that in the WT, while the content of As in the grains was higher than that of the WT [42]. OsABCC7 was involved in the transport of As(III) from the roots to the shoots. The knockout of *OsABCC7* significantly reduced the concentration of As in the xylem sap but had no significant effect on the content of As in the roots [44].

Currently, most of the research on the metabolism of As in plants focuses on the pathway of As(III) transport from the root to the shoot, and the redistribution of As(III) in different tissues of the shoots at different growth stages merits further exploration. In addition, the existence of As(III) in different parts of the rice grains and the influencing factors of its distribution merits further clarification.

## 4. Absorption, Translocation and Redistribution of As(V) in Rice

### 4.1. Absorption of As(V)

Under drought or oxidation conditions, As(V) is the primary species of As [31]. Since As and P belong to the fifth group of the periodic table, and the oxidation state of As(V) and phosphate are both +5, the chemical properties of As(V) and phosphate are very similar, As(V) is a chemical analog of phosphate (Pi), rice primarily absorbs As(V) through a variety of Pi transporters. The presence of As(V) and Pi deficiency in the growth environment will enhance the co-transport of As(V) and Pi [33]. Pi transporters, such as OsPT1 [66], OsPT4 [67] and OsPT8 [68], TFs, such as OsPHR2 [69] and OsWRKY28 [70], and the Pi transporter transport promoting factor OsPHF1 play a key role in the absorption and transport of As(V) in rice [69] (Table 2). A total of 13 Pi transporters (OsPT) are involved in the transport of As(V) in the rice roots [71,72]. As(V) primarily enters the root cells of rice through OsPT1 [66], OsPT4 [67] and OsPT8 [66,68]. Kamiya et al. (2013) found that the concentration of As in the *ospt1* mutant shoots was 60% lower than that of the WT [66]. The absorption of As by the *ospt4* mutant decreased, and the As(V) content in rice roots was 50–55% lower than that of the WT [67], while the accumulation of As(V) in the *OsPT4* overexpression lines increased [67]. The Pi transporter OsPT8 is also involved in the absorption of As(V) in rice. The As(V) content absorbed decreased by 33–57% in the *OsPT8* mutation line and increased 3- to 5-fold in the overexpressed *OsPT8* line [68]. In addition, the tolerance of rice to As(V) stress increased 100-fold [68]. OsPT2, OsPT6 and OsPT11 are involved in the absorption of Pi in rice roots and indirectly affect the absorption of As(V) [73,74]. The TFs OsPHR2 and OsPHF1 are also involved in the absorption of As(V) [69]. Studies have found that 60~90% of the As(V) absorbed by rice will be excreted from the roots in the As(III) form [75].

Is there a specific transporter that mediates the absorption of As(V)? The type, quantity and regulation of Pi transporters significantly affect the absorption of As(V). Can the transporters that inhibit the absorption of P also inhibit the absorption of As(V)? The structure and chemical properties of antimony, P and As are similar. Will antimony transporters also transport As like Pi transporters? Subsequent research can focus on these scientific issues.

### 4.2. Transport of As(V)

Except for a small amount of As(V) that accumulates in the outer epidermis of the root, the remaining As(V) is loaded into the xylem. As(V) is reduced to As(III) by arsenate reductases, such as OsHAC1;1 [76,77], OsHAC1;2 [76], and OsHAC4 [78], in the roots of rice. Therefore, As(V) reductase affects the transfer and accumulation of As in rice. The knockout of the arsenate reductase *HAC1* (High As Content 1) gene inhibited the activity of arsenic reductase, which resulted in a decrease in the reduction of As(V) and As(III) efflux, and increased the accumulation of As in the shoots [76,78]. As(V) is transported from the roots to shoots through the xylem [83]. The content of As(V) in the shoots of the *ospt1* knockout mutant was 60% lower than that of the WT [66], while the overexpression of *OsPT1* promoted the accumulation of As(V) in the shoots of rice [66]. OsPT8 significantly increased the transport of As(V) to the shoots [69]. The content of inorganic As in the grains of *ospt4* mutant was significantly reduced [67], and the transport coefficient of As(V) from the roots to shoots decreased by 33.3–39.6% when *OsMATE2* was constitutively overexpressed in tobacco (*Nicotiana tabacum*) [82]. As(V) stress decreased the levels of expression of *OsPT8*, *OsPT4* and *OsPHO1;2* in the rice roots, while it increased the level of expression of *OsPCS1* and the content of PCs, indicating that As(V) stress enhanced the compartmentation of As in the vacuole of root cells, thereby limiting the transport of As(V) to the shoots [24].

Will As speciation change during the transport of As(V) to the shoot of rice? If so, when and where? What is the cause? Do nodes regulate the long-distance transport of As(V)? These issues merit further study.

### 4.3. Redistribution of As(V)

A total of 56% of As(V) was transported to the grains through the phloem [84], and it was primarily enriched in the vascular bundles of caryopses [64,85]. The level of expression of *OsMATE2* in the developing seeds under As(V) stress increased 6-fold, which positively correlated with the content of As in the mature grains. The specific silencing of *OsMATE2* in the rice endosperm reduced the content of As in the grains by 36.9–47.8% [82].

There are few studies on the manner, mode and regulation of the redistribution of As(V) in the shoots of rice. In the future, the path by which As(V) enters the grain and the manner and mechanism of As(V) enrichment in different parts should be further clarified to lay a theoretical foundation for reducing the accumulation of As(V) in the grains.

## 5. Absorption, Transport and Redistribution of Organic As in Rice

### 5.1. Absorption of Organic As

Under the catalysis of S-adenosylmethionine methyltransferase (arsM), As(III) can be gradually methylated into mono, di and trimethyl arsenite [86,87]. Flooding and hypoxic conditions also enhance the microbial methylation of As [88], such as by sulfate-reducing bacteria involved in the methylation of As [89]. DMA and MMA enter rice through OsLsi1 as an undissociated species [90] (Table 3). The absorption of MMA and DMA by the *oslsi1* mutant was 80% and 49% lower than that of the WT, respectively, while the *OsLsi2* mutation had no significant effect on the absorption of MMA and DMA [90]. The aquaglyceroporins, a sub-class of aquaporins that facilitate the diffusion of water, glycerol and other small uncharged solutes across cell membranes [91] on the plasma membrane promote the absorption of DMA and MMA in the roots of rice [92]. Although DMA and MMA have the same pathway of absorption as As(III), the rates of absorption and efficiency of organic As were lower than those of inorganic As [93,94]. The absorption or average root absorption factor of As(V) was fivefold higher than that of DMA and 2.5-fold higher than that of MMA. In addition, MMA(V) was partially reduced to MMA(III) in the rice roots [90], but the mechanism merits further study.

In addition to DMA and MMA, does rice absorb other species of organic As? In addition, whether organic As, particularly volatile As, will be absorbed by aboveground organs, such as the leaves, still merits further clarification. Currently, few organic As absorption transporters have been reported, and other new transporters should be further identified and excavated.

### 5.2. Transport of Organic As

Compared with the other species of As, DMA is more easily transferred from the rice roots to shoots [71]. The concentration of As in the rice grains, stems and leaves treated with DMA was more than 100-fold higher than when inorganic As was used [96]. The transport of organic As in rice is more efficient and quicker than that of inorganic As, which may be due to the fact that organic As forms fewer complexes with glutathione (GSH) or phytochelatins in the roots [93] or is in a good dissociation state under cytoplasmic pH conditions [97]. The organic As was transported to the xylem of rice through OsLsi1 and then transported upward [90]. Only MMA(V) and not MMA(III) were transported to the shoots [90]. The concentrations of MMA and DMA in the shoot and xylem sap of the *oslsi1* mutant were lower than those of the WT, while the concentrations of MMA and DMA in the same part of the *oslsi2* mutant did not differ significantly from those of the WT [90], which indicated that OsLsi1 rather than OsLsi2 is involved in the transport of organic As to the shoots. In addition, the peptide transporter OsPTR7 in rice is involved in the long-distance transport of DMA [95].

Similar to inorganic As, the transport of DMA or MMA to the shoots is also related to the valence state of As, and the reason merits further exploration. Are the As species transformed during the long-distance transport of DMA or MMA? Whether it will be converted into volatile As species and then discharged from rice merits further clarification.

### 5.3. Redistribution of Organic As

Rice cannot methylate As in vivo, and the organic As in rice originates from the soil [88]. In rice grains, methylated As primarily exists as a species of DMA and partly in the species of MMA and trimethylarsine oxide (TMAO) [98,99]. A total of 55% of the DMA was transported from the leaves to grains [100], and a total of 100% of the MMA(V) and 89% of the DMA(V) were transported to grains through the phloem [84]. OsPTR7 is involved in the accumulation of DMA in the rice grains, and the knockout of this gene reduced the amount of DMA available for long-distance transport to the grains [95]. In addition, DMA is more likely to enter the endosperm [64,85], while the route of MMA remains unclear. The distribution of DMA or MMA in the aboveground tissues, particularly the distribution of MMA in grains and its molecular mechanism, merits further study.

## 6. The Primary Strategies to Reduce the Accumulation of As in Rice

**Figure 2:**
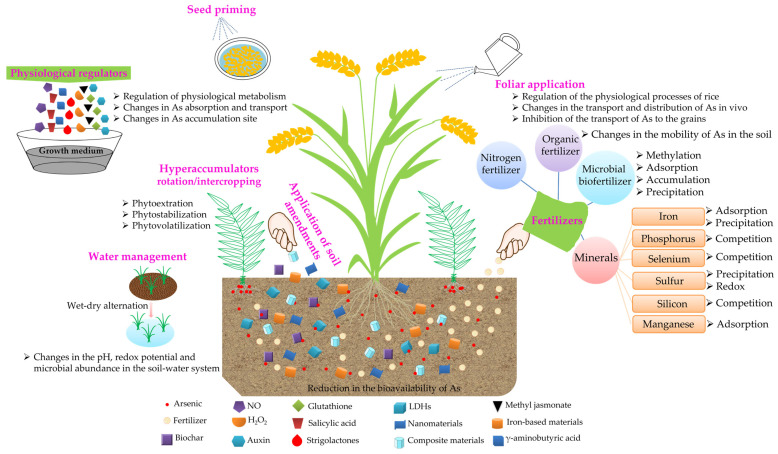
Agronomic measures to reduce the accumulation of arsenic in rice.

Currently, the primary strategies to reduce the accumulation of As in rice are divided into two categories, including agronomic practices and biotechnology. The agronomic practices primarily include the use of iron [85], P [101], sulfur (S) [102], silicon (Si) [103], selenium (Se) [104] and manganese (Mn) [105] to supplement the soil; the application of amendments; the development of water management; foliar spraying, and the use of organic fertilizers and microbial biofertilizers among others to reduce the effectiveness of As in the soil, change the metabolism and transport methods of As to reduce its absorption by rice and accumulation in grains. In addition, the application of physiological regulators in the growth media, inoculation with microorganisms that hyperaccumulate As, rotation or intercropping of the rice with As hyperaccumulators, the application of nitrate nitrogen fertilizer and seed priming techniques can also reduce the content of As in rice (Figure 2). Biotechnological approaches include breeding rice varieties that accumulate low levels of As by hybridization or changing the patterns of expression of the genes involved in the absorption, transport and metabolism of As through transgenic methods to create new germplasm resources.

### 6.1. Agronomic Practices

The agronomic measures to reduce the accumulation of As in rice are primarily by reducing the absorption and transport of As by changing the bioavailability of As in the soil–water–rice system, the expression of transporter genes, and the activity of mineral elements.

#### 6.1.1. Application of Minerals

##### Iron

The application of iron can reduce the availability of As in the soil solution [101], reduce the content of total and inorganic As in the roots and grains [106], and reduce the transport factor index of As in the roots/soil, stems/roots and grains/stems [107]. Under certain conditions, the addition of iron to the soil can produce iron hydroxides (Fe oxyhydroxides [FeOOH]) [108,109,110], which can promote the adsorption of As. Secondly, the addition of iron can form an iron plaque or the deposition of iron oxide in the roots of rice. The iron plaque has a high affinity for As, which, in turn, adsorbs more As, thus resulting in a decrease in the availability of soluble As in the paddy field [111]. The absorption and transport of As to the shoot are then reduced [111]. The formation of iron plaque is also conducive to the sequestration and adsorption of As through radial oxygen loss and rhizosphere microbial activities, which results in a decrease in the absorption of As and its transport and accumulation [17]. When iron is applied, it is necessary to consider the amount of iron used to prevent the iron plaque from being too thick and negatively affecting the absorption of nutrients and diffusion of oxygen [112]. It is also necessary to consider the soil organic matter (OM), physical and chemical properties, and the growth stage of rice. These factors will regulate the absorption of As by affecting the properties of the solution of iron and soil. For example, the combined treatment of ferrous sulfate, farmyard manure and vermicompost significantly reduces the concentration of exchangeable As [113].

The multiple valence states and existence of forms of iron and the complexity and dynamics of the soil environment increase the difficulty of accurately determining the amount of iron applied that can effectively reduce the absorption of As. In practical applications, the key growth period can be selected in combination with flooding management to maintain the reduction state of paddy soil to stabilize the concentration of ferrous iron (Fe^2+^).

##### Phosphorus

The addition of P to the soil reduced the content of As in rice, primarily because the absorption, transportation and metabolism of As(V) in rice are conducted by the same transporter as P, and the increase in the concentration of P in the soil competitively inhibited the absorption of As(V) [114]. The application of P significantly reduced the negative effects of As on the agronomic traits, such as root weight, root length, plant height and grain yield, and the application of 30 mg kg^−1^ of P could minimize the accumulation of As in rice grains [115]. The content of As in the rice roots decreased with an increase in the concentration of external inorganic P, and the accumulation of As in grains significantly negatively correlated with the content of P in the roots (r = −0.25 *) [116]. The application of P to the soil requires consideration of the amount of Pi and soil properties. The addition of P does not necessarily inhibit the absorption and accumulation of As in rice [117], and it can even increase the total concentration of As in rice plants and grains [118,119]. The primary reason is that orthophosphate (PO_4_^3−^) and As(V) are competitively adsorbed on the soil matrix and root iron plaque. The excessive addition of P easily replaces As in the soil through a ligand exchange mechanism [120]. As is desorbed from the mineral surface [121], which results in an increase in the concentration of As(V) in the soil solution and an increase in the bioavailability of As in the rhizosphere [118]. The application of P fertilizer in soil that is deficient in P can cause the adsorption of P at the soil exchange sites, thereby increasing the bioavailability of As [122]. In soils with low P that have been contaminated with As, amendment with P can lead to an increase in the bioaccumulation of As [123].

The excessive application of P can lead to the pollution of rivers, groundwater and other bodies of water. Under drought conditions, As(V) comprises a relatively high proportion of the soil, and the application of P can be more effective at reducing the concentrations of As. Therefore, on the premise of ensuring the normal growth of rice, the application of P fertilizer to regulate the absorption of As by rice should be conducted where the content of water in the soil water is minimal.

##### Sulfur

The mechanism of reducing the absorption of As by rice by adding S is primarily used because S reduces the availability of As and participates in the redox and detoxification processes of As in rice. First, the addition of S can reduce the accumulation of As in rice by changing the mineral structure of the rhizosphere. Under flooding conditions, sulfate ion (SO_4_^2−^) in the soil–water system is reduced to sulfide ion (S^2−^) [124], and As(III) can react with S^2−^ and precipitate it as arsenic trisulfate (As_2_S_3_) or arsenic sulfide (AsS) [125,126], thereby reducing the bioavailability of As(III). In the rhizosphere, thiols and other compounds that contain S limit the availability of As in the soil solution by chelating with As [102]. Sulfur induces the formation of iron plaque in the rhizosphere and root surfaces, which hinders the absorption of As by rice [127]. Secondly, S can also promote the formation of PC and GSH in the rice roots [102], combine with As(III) to form As(III)-thiol complexes and isolate them in vacuoles [124]. The application of S fertilizer significantly reduced the accumulation of As in the rice leaves [128]. Duan et al. (2011) found that the foliar application of L-buthionine-sulphoxime reduced the accumulation of As in the shoots by regulating the biosynthesis of GSH and PC [129]. In addition, when S fertilizer is applied, the formation of thioarsenic species should be monitored [130].

Whether the effects of different types of S fertilizer and spraying methods on the regulation of As accumulation in rice are similar merits further study. Simultaneously, the precipitation and redox of sulfur are related to the soil pH and oxidation/reduction potential (Eh), and the combination of S fertilizer with water results in better control of As.

##### Silicon

Silicon can compete with As(III) to enter the rice plants and reduce their absorption of As(III) [103]. Simultaneously, an increase in the concentration of Si inhibited the expression of Si transporters, thereby reducing the accumulation of As [45]. The increase in Si/As(III) ratio in the rhizosphere was the key factor in reducing the absorption of As(III) by rice. With the increase in additional Si in paddy fields contaminated with As, the ratio of As(III) to total As in the rice tissues decreased significantly [131,132]. The addition of Si significantly reduced the concentration of inorganic As [primarily As(III)] in the vegetative and reproductive organs of rice but increased the concentration of DMA in the rice grains [133]. Those who apply Si fertilizer need to consider the amount and type of application [134,135] and the soil particles. In addition, the use of Si-based fertilizers or Si mineral complexes can reduce the xylem loading, transportation and local accumulation of As [136]. However, the use of commercially synthesized Si fertilizers is expensive for small-scale farmers. In view of the high content of Si in rice plants, which can comprise 10% of the dry weight of the shoots [137], the straw and rice husk can be composted and returned to the field as a raw material to serve as Si fertilizer, and the appropriate period of fertilization and method can be selected to reduce the amount of ineffective fertilization.

##### Selenium

The primary mechanism of the reduction in As by Se is that the channels of Se and As that enter the rice overlap, which can cause antagonism between the two elements [104]. After supplementation with Se, the plants can repair the structural deformities in the wall and disintegrate of cell membrane induced by As. Compared with As exposure alone, the activities of S-transferase (GST) and glutaredoxins (GRX) are increasing, so there is a stronger scavenging effect on free radicals produced by As [29] and the levels of expression of the genes related to As transport, such as *NIP1;1*, *NIP2;1*, and *NRAMP1*, and the sulfate transporter genes, such as *SULTR3;1* and *SULTR3;6*, increased in the As + Se treatment. In addition, the levels of expression of regulatory factors, such as AUX/IAA, WRKY and MYB, were also higher, and the oxidative stress induced by As was alleviated, thereby reducing the accumulation and toxicity of As [29], and helping to reduce the reduction in yield caused by As toxicity. Selenite regulates the levels of phenolics and nutrients in rice, while selenate improves the contents of amino acids and thiol metabolism, thereby alleviating the toxicity of As(III) [138,139]. In addition, both selenite and selenate facilitate the adsorption of As(V) by the soil, thereby reducing the absorption of As(V) by the rice roots. Selenate is more effective at inhibiting inorganic As transfer factors than selenite, indicating that various forms of selenium can reduce the toxicity of As to rice to varying degrees. In actual production, factors, such as the type of Se fertilizer, method of application and cost of production should be comprehensively considered.

##### Manganese

Manganese oxides are naturally strong oxidants and scavengers that can regulate the mobility and bioavailability of As [105]. The primary mechanism of the addition of Mn to reduce the content of As is by forming Mn plaque in the roots, which limits the absorption of As by adsorbing Mn. For example, in paddy fields contaminated with As, the total concentration of As in the straw and grain can be reduced by 30–40% by applying synthetic Mn oxides (hausmannite) at a ratio of 1200 mg Mn kg^−1^ soil [140]. The α-MnO_2_ nanoparticles reduced the content of available As, increased the content of residual As and the insoluble binding As (Ca-As and Fe-As), promoted the oxidation of As(III) to As(V), increased the adsorption of As onto indigenous iron (hydr)oxides, and significantly reduced the content of As in the water in soil pores [105]. With the increase of Mn in the soil, the accumulation of As in brown rice decreased [105]. However, studies have also found that additional Mn cannot adsorb As in paddy soil [141], and excessive Mn will be toxic to the rice plants [142]. Therefore, when Mn is applied, it is important to note the differences in dosage and rice varieties [143]. In addition, the soil was in a reduced state after flooding, and the As and mineral elements originally adsorbed on the Mn plaque were easily desorbed with the reduction of manganese oxides, which resulted in an increase in the concentration of As and mineral elements in the soil solution. In view of the complexity of the paddy soil environment, the foliar spraying of Mn fertilizer at key growth stages can be considered as a method to reduce the accumulation of As in rice.

#### 6.1.2. Application of Soil Amendments

Amendments include modified biochar, modified iron-based materials, nanomaterials, composite amendments and layered double hydroxides (LDHs, also known as hydrotalcite) among others. The primary mechanism is to passivate the bioavailability of As by changing the state of occurrence of As in the soil, thereby reducing the absorption of As.

##### Modified Biochar

As a new, environmentally friendly and renewable carbon material, biochar is widely used to remove As from soil and water due to its excellent capacity for adsorption and low cost [144]. To improve the efficiency of adsorption, biochar-based materials have been modified by single metal(oid) or its salt, such as Si [145], zerovalent iron [146], black iron oxide (Fe_3_O_4_) [147], and aluminum chloride (AlCl_3_) [148] among others, or multiple metal(oid)s or/and their salts, such as Ce-Mn [149] or Fe-Mn-La [150]. The incorporation of rice husk biochar rich in Si resulted in a 30% decrease in the content of inorganic As in grains [145]. The addition of sewage sludge biochar reduced the concentrations of As(III), As(V) and DMA in rice by 72%, 62% and 74% in the grain, respectively [151], which could be due to the addition of biochar to promote the formation of volatile As. However, some types of biochar can also lead to an increase in the content of As in rice plants and grains [152], which may be related to the microbial action on biochar or a change in the soil properties [152]. Therefore, there have been studies on the surface modification of biochar, such as the use of zero-valent iron nanoparticles or modified Mn biochar, to reduce the accumulation of As in the rice tissues [146,152,153]. A new type of calcium-based magnetic biochar (Ca-MBC) was synthesized by the pyrolysis of rice straw, Fe oxide and calcium carbonate. The iron oxide on the surface of Ca-MBC can form monodentate and bidentate chelates with As, and these chelates can strongly adsorb As. Compared with the activation of As by ordinary biochar, Ca-MBC increases the proportion of residual As in the soil to 97%, which results in a significant reduction in the content of bioavailable As [154].

Currently, the influencing factors and mechanism of the remediation of As-contaminated soil-modified biochar still remain unclear. It is necessary to deeply reveal the micro-mechanism of modified biochar on the immobilization of As in the soil and lay a theoretical foundation for the development of mature, stable and efficient remediation technology.

##### Modified Iron-Based Materials

Both Fe-Ca composite (FeCa) and Fe-Mn binary oxide (FMBO) are effective at stabilizing As in the soil. When the same amount of these two iron-based materials is added, FeCa is more effective than FMBO at stabilizing the As in soil, and the efficiency of stabilization significantly negatively correlates with the content of clay in the soil [155]. When iron-based biochar was applied at 1.5 and 3.0 t·ha^−1^ as an amendment in a rice–wheat rotation farmland, the available As in the rice soil decreased by 15% and 12% in the first year, respectively, and the content of As in the grain decreased by 37% and 25%, respectively. The proportion of As in the soil that was available for reduction in the second year did not change significantly compared with the first year, but the content of As in the grains decreased by 57% and 61%, respectively [156]. The mechanism of immobilization of As by iron-based biochar primarily includes the following: (1) the release of iron ions or iron (hydrogen) oxides from biochar into the soil to form FeAsO_4_-H_2_O and FeAsO_4_-2H_2_O complexes or precipitates [157]; (2) the possible oxidation of Fe^2+^ to ferric iron (Fe^3+^), which can form more ferrihydrite, goethite or hematite to enhance the adsorption of As [158]; and (3) the presence of iron promoted the formation of many iron plaques on the root surface, which facilitated the fixation of As and reduced the absorption of As in the roots [159].

The mechanism of action of the iron-based materials on As in the soil is more complex than that of a single iron element or iron compound, and many factors need to be considered to study its effect at stabilization. It is likely to be difficult to succeed in the successful application of iron-based materials during the practice of production in the future.

##### Nanoparticles

Nanoparticles, which are composed of metal/metalloid or metal/metalloid oxides, include manganese dioxide (MnO_2_), zinc oxide (ZnO), copper oxide (CuO), cerium oxide (CeO_2_), titanium dioxide (TiO_2_), manganese dioxide (MnO_2_), iron oxide (magnetite [Fe_3_O_4_]) and maghemite (Fe_2_O_3_)], alumina, Si, silicon dioxide (SiO_2_) and Se among others [160]. It primarily uses the characteristics of nanoparticles, which include their small size, large specific surface area, strong adhesion ability and slow release of active substances. Nanoparticles enter the soil and can be adsorbed by soil particles to form aggregates or degraded by indigenous soil microorganisms [161]. The application of MgO nanoparticles activates the antioxidant defense system and significantly reduces the accumulation of ROS by hindering the transport of As from the soil to rice plants [162]. Compared with rice treated with As alone, the application of ZnO nanoparticles significantly reduced the total content of As in the roots and shoots [163]. The application of CuO nanoparticles reduced the contents of As(V) in the roots and shoots of rice by 54% and 55%, respectively, while As(III) caused a decrease of 23% and 45%, respectively [164]. The application of MnO_2_ nanoparticles reduced the total concentration of As in the grains by 65.4% [165]. The total content of As in the grains decreased by 28% and 40% in pot and field experiments with the application of silica nanoparticles to the soil, respectively [166]. The application of nano-Fe_3_O_4_ and nano-zerovalent iron reduced the transport of As from the roots to shoots, and the effect was better than that of high-quality graphene oxide, multilayer graphene oxide, and 20 nm or 40 nm of hydroxyapatite [167]. A volume of 1% nanoscale zero-valent iron material augmented with 1% zeolite could reduce the available As in the soil by 91.6% [168]. With the application of TiO_2_ nanoparticles, the rice seedlings grew normally, while the accumulation of As decreased by 40–90% [169]. CeO_2_ nanoparticles significantly reduced the content of As(V) in the roots of rice seedlings, but it had no significant effect on the content of total As under As(III) stress [170], which could be due to the combined application of CeO_2_ nanoparticles and facilitation of the release of root exudates due to the presence of As(V), which provides electrons to reduce As(V) to As(III) [171]. Selenium nanoparticles significantly reduced the contents of total As, As(III) and As(V) in the shoots of rice, which alleviated the toxicity of As [172].

Currently, there are increasing types of nanomaterials, and they have varying mechanisms such as adsorption [160], redox reaction [162,166,169], coprecipitation [168], etc., to reduce the absorption and accumulation of As in rice. The selection of nanomaterials should be combined with the local features. In addition, most nanomaterials currently use metals and metalloids as basic raw materials, and their potential effects on the soil properties, rice growth and ecological environment merit further clarification. The use of synthetic nanoparticles for plants is an environmentally friendly restoration material with broad prospects for application.

##### Composite Amendments

The use of composite amendments (iron sulfate + poultry manure + modified biochar) significantly reduced the content of exchangeable As in the soil, while the content of As bound to Fe-Mn or OM, and residual forms of As has a tendency for an increase [173]. The application of a composite modifier (hydroxyhistidine + zeolite + biochar) promoted the transformation of soluble As to insoluble As bound to calcium. The content of As bound to calcium increased by 2.8–53.3%, while the content of exchangeable As decreased by 7.0–39.5%. When the rate of application was 0.5~2.0 g kg^−1^, the content of As in the aboveground organs of rice could be reduced. An application rate of 2 g kg^−1^ reduced the content of As in brown rice < 0.2 mg kg^−1^, which reached the national standards for food pollutants. When the rate of application > 4.0 g kg^−1^, the content of exchangeable As increased, and there was a decrease in the enrichment of As in the rice roots [174]. Composite amendments were more effective at reducing the accumulation of As in rice than a single component, which could be due to the additive relationship of various components. Thus, this resulted in the universal use of composite amendments.

##### LDHs

LDHs, one type of layered materials and also known as hydrotalcite-like systems or anionic clays [175] are considered to be ideal materials to adsorb As because they have a large specific surface area and are porous [175]. Compared with other heavy metals, such as lead (Pb) and molybdenum (Mo), LDHs are more effective at adsorbing As; this is particularly true for As(V) [176]. The rates of removal of As(III) and As(V) by ZnAl-SO_4_-LDHs were 69% and 88%, respectively [177]. CaMgFe-LDH is more effective at removing As(III) and immobilizing As than MgFe-LDH, which could be due to the dual effects of Ca^2+^ precipitation released by the LDH matrix and interlayer anion exchange. Currently, the material has been successfully applied to paddy field systems that were contaminated with As [178].

#### 6.1.3. Application of Fertilizers

##### Nitrogen Fertilizer

The application of nitrate fertilizer inhibited the reduction and dissolution of iron minerals that contained As in flooded soil, which enhanced the oxidation of As(III) mediated by microorganisms and reduced the bioavailability of As in the soil. The total concentration of As and ratio of inorganic As to total As in the grains decreased by 32.4% and 15.4%, respectively [179]. Another study found that under flooding conditions, the application of nitrate nitrogen fertilizer in soil contaminated with As reduced the total accumulation of As in different parts of the rice plants by approximately 40% [180]. The application of nitrate nitrogen fertilizer is more convenient and does not increase the cost of production. It can not only reduce the accumulation of As in rice but also promote the growth of rice and increase its yield, which is a strategy that is consistent with the actual production. However, the application of ammonium nitrogen in flooded soil promotes the reduction and dissolution of iron minerals that contain As and promotes the reduction of As(V), which is mediated by microorganisms. This results in the release of As from the soil to pore water. The application of ammonium nitrogen fertilizers, such as urea and ammonium bicarbonate, resulted in a significant increase in the ratio of As concentration to the adsorption of inorganic As [179]. In addition, the simultaneous application of ammonium nitrogen and nitrate nitrogen fertilizer had no significant effect on the concentration of As and its transformation in paddy fields [179]. It is apparent that different forms of N fertilizers and application methods affect the species of As and its total As in rice. Thus, the types of N fertilizers that are applied to paddy fields contaminated with As should be screened.

##### Organic Fertilizer

Organic fertilizer primarily provides OM to the soil, thereby increasing or reducing the mobility of As in the soil [123,181]. When organic matter is applied, the As content in soil markedly decreased, especially with farmyard manure application [182]. An increase in the amount of OM in the soil can promote the growth of microorganisms and create favorable reduction conditions, which results in the reductive dissolution of ferric hydroxide, a decrease in the soil redox potential and an increase in the mobility of As [183,184]. The positive correlation between OM and the mobility of As could lead to an increase in the content of As in the grains [185,186]. However, some studies have found that the OM significantly negatively correlated with the accumulation of As in grains [71,114,187,188]. In addition, since OM can increase or decrease the mobility of As in the soil, OM should be applied with caution in soils that contain high concentrations of As to prevent its accumulation in rice.

##### Microbial Biofertilizer

The use of certain bacteria, fungi or plants or even whole microbial-plant bioremediation to remove excessive As in paddy fields is a feasible method to reduce the concentration of As in contaminated soil [189]. Microorganisms can cause the morphological transformation, movement and passivation of As by methylation, adsorption, and accumulation and precipitation [190]. Soil microorganisms convert inorganic As into less toxic organic As, or they oxidize As(III) to As(V) under semi-dry conditions. Thus, this changes the bioavailability of As or dilutes its concentration by increasing the plant biomass, which plays an important role in reducing the accumulation of As [191,192,193]. Bacterial activity promoted the transformation of As species, and the soluble exchangeable As decreased from 50 mg kg^−1^ to 0.7 mg kg^−1^ after 7 days of treatment [194]. Microbial fuel cells can utilize the bio-catalytic capabilities of viable microorganisms and use a range of organic fuel sources by converting the energy stored in the chemical bonds to generate an electrical current instead of relying, for example, on the use of metal catalysts [195]. The application of microbial fuel cells in paddy fields can reduce the accumulation of As in grains [196]. In addition, microorganisms that can promote rice growth include bacteria, microalgae (cyanobacteria) and vesicular arbuscular mycorrhizae (VAM) fungi that can potentially affect the absorption of As in rice. For example, *Brevundimonas diminuta* NBRI012 is an As-resistant strain of rhizobium that can produce the plant hormone indole acetic acid (IAA), a type of auxin, which can effectively reduce the toxicity of As and promote the growth of rice [197]. Vesicular arbuscular mycorrhizal fungi reduce the absorption of As(III), As(V) and MMA by inhibiting high- and low-affinity P absorption systems [198], thereby reducing the ratio of inorganic/organic As in rice [199]. Phytochelatins (PC) are thiol-rich peptides having the general formula γ(Glu-Cys)n-Gly, where n ranges from 2 to 11 (called accordingly as PC2, PC3, PC4, etc.), produced in plants in response to stress from heavy metals and metalloids including As [200]. Some algae can synthesize PCs and metallothioneins, which can form complexes with the absorbed As and transport them to the vacuoles for storage [201]. The inoculation of soil with *Chlorella vulgaris* Beijerinck and *Nannochloropsis* sp. can reduce the inhibition of rice growth caused by As toxicity, and the absorption of As was reduced by 30–40% [23]. Microbial biofertilizers, which can reduce the accumulation of As in rice, have attracted increasing amounts of attention from researchers and practical use by producers due to their high efficiency, low cost and minimal health hazards.

#### 6.1.4. Foliar Application

Foliar applications change the transport and distribution of As in rice by spraying physiological antagonists or physiologically active substances on the leaves to prevent As from being transported to the grains. Currently, most of the primary foliar inhibitors that are applied include inorganic compounds, such as P, S, Si, Se, Fe, Mn, and zinc (Zn) among others. The foliar application of Si to the leaves regulated the contents of trace elements, antioxidants and thiols in rice, which inhibited the transport of As and decreased the content of As in grains [202]. In addition, the foliar application of Si increased the thickness and mechanical force of the rice cell wall and its content of pectin and activity of pectin methylesterase (PME) in the cells; it significantly down-regulated the levels of expression of the genes *OsLsi1* and *OsLsi2* in the roots and *OsLsi6* in the leaves and sheaths that are related to the adsorption and transport of As, thereby reducing its transport and accumulation to the grains [203,204]. The foliar application of silica nanoparticles significantly increased the concentration of As in the nodes and limited the migration of As to the leaves and grains, which resulted in a decrease in the concentration of As in the grains by 7.11–15.1% [205]. However, some studies have found that the foliar application of Si did not effectively reduce the accumulation of As in rice seedlings [206]. The foliar application of 2,3-dimercaptosuccinic acid to rice leaves at the flowering stage resulted in a decrease in the contents of total and inorganic As in brown rice by 36.4% and 51.2%, respectively, while the content of As in the leaves increased by 32.67%, indicating that more As was transported to the leaves, thereby reducing the content of As in the edible parts [207]. The application of 15 mg·L^−1^ chitosan-based Si nanoparticles alleviated the damage of lipid peroxidation in rice, increased the activity of CAT and the content of GSH, and protected the plants from oxidative stress by changing the contents of sulfhydryl groups and disulfide bond complexes of the membrane proteins. Simultaneously, GSH promoted the formation of PCs and reduced the migration of As, which resulted in an increase in the content of As in the husks by 61.2% and a decrease in the content of As in brown rice by 35.4% [208]. The foliar spraying of chitosan-based Si nanoparticles promoted the fixation of As in the cell walls of leaves, which resulted in a decrease in the concentration of As in the grains by 61.2% [208].

Since the rice leaves are covered with a waxy layer, 95% of the foliar inhibitors cannot enter the plant from direct spraying [209]. In addition, high concentrations of inorganic solutions of foliar inhibitors can also burn the leaves and reduce their photosynthetic efficiency, while low concentrations of foliar inhibitors are often ineffective. Therefore, the development of effective inhibitors that can be absorbed by leaves is the focus of future research and application.

#### 6.1.5. Water Management

As moves more easily and is more effectively absorbed under flooded conditions. Therefore, modifying the manner in which the rice is irrigated to meet its normal needs for growth can be an effective way to reduce the accumulation of As in rice, such as practicing intermittent irrigation and aerobic water management to maintain the aerobic environment in the soil [26], and the rice production in intermittent flooding treatment was higher than in continually flooding treatment [210,211,212,213,214], or non-significantly affecting crop yield [215]. Changes in the properties of the soil–water system, such as the pH, redox potential and microbial abundance, can affect the absorption and transport of As by regulating the bioavailability of As, the levels of expression of the transporter genes and the activity of mineral elements [26,113,212,216]. Under aerobic conditions, the concentrations of As in the grain and straw decreased 10- to 20-fold and 7- to 63-fold, respectively [90]. When the soil is severely dry, it not only reduces the accumulation of As in grains but also affects the species of As [217]. The application of an oxygen-releasing compound (ORC), such as calcium peroxide (CaO_2_), under flooded conditions in paddy fields can reduce the accumulation of As in rice, but it is not suitable for high concentrations of As in a stress environment [218]. Ma et al. (2020) found that the application of 10 mg L^−1^ of nitrate and/or 50 µg L^−1^ of perchlorate (ClO_4_^−^) in the irrigation water reduced the content of total As in the grains by 34–45% and increased the yield of rice by 35–93%, while the application of perchlorate alone led to the highest decrease in total As [219]. In addition, compared with the flooded soil, the physical and chemical parameters of the soil changed more frequently under alternating dry–wet environments, and the microbial community was more abundant and diversified. The bioavailability of soil As decreased under the influence of biotic and abiotic factors [216]. A full understanding of the characteristics of As absorption and the water demand of rice at different growth stages and adopting appropriate measures of water management during each growth stage can not only maintain the normal growth of rice but also reduce the accumulation of As in vivo.

#### 6.1.6. Others

##### Physiological Regulators

The application of some physiological regulators to the culture medium resulted in a reduction in the accumulation of As by regulating the physiological and biochemical metabolism of rice, thereby reducing the toxicity of As. The exogenous application of γ-aminobutyric acid (GABA) can increase its endogenous level, induce the expression of GABA shunt genes, inhibit the accumulation of ROS, and reduce the levels of expression of *OsLsi1* and *OsLsi2*, which resulted in a decrease in the accumulation of As in rice seedlings [220]. The exogenous application of nitric oxide (NO) significantly reduced the accumulation of As in the roots and shoots, inhibited the transport of As from the roots to shoots, regulated antioxidant and thiol metabolism [221,222], and induced the levels of expression of *OsGRX9* and *OsGSTs* [223]. When the rice was exposed to As(III) stress, methyl jasmonate could reduce the content of As(III), which resulted in a decrease in the contents of MDA and rate of electrolyte leakage, increase in the content of chlorophyll, enhanced activities of the antioxidant enzymes, up-regulation of the expression of *OsPCS2*, and an improvement in the tolerance of rice to As [43]. The application of GSH increased the activity of guaiacol POD and SOD in sensitive and resistant varieties of rice under As stress and enhanced the tolerance of rice to As [224]. Treatment with salicylic acid (SA) had no significant effect on the accumulation of As in the roots under As(III) stress [221], while the accumulation of As in the shoots decreased by 27% [221]. In addition, under As stress, the exogenous application of SA promoted the biosynthesis of NO [221]. The SA interacted with ethylene and NO, promoted the photosynthesis and growth of rice, regulated the antioxidant defense system, and enhanced the tolerance to As [225]. The application of H_2_O_2_ protected the photosynthetic activity of rice from As toxicity by regulating the activity and expression of the antioxidant enzymes [226]. The addition of auxin (IAA) increased the length, fresh weight, and the contents of chlorophyll, protein and cysteine in the rice shoots under As stress, which reduced the content of MDA and alleviated the toxicity of As [227]. Strigolactones (SLs) are involved in alleviating As stress by regulating the As(V) absorption, vacuole compartments, biosynthesis of GSH and antioxidant defense responses in rice [228]. An analysis of the physiological metabolic pathways of rice in response to As stress and clarification of the key regulatory factors facilitate the accurate selection of physiological regulators.

##### Hyperaccumulators

The use of rotations and the intercropping of hyperaccumulators with rice reduced the accumulation of As in rice decreasing the content of As in the soil [189,229]. Chinese brake (*Pteris vittata*), maidenhair fern (*Adiantum capillus-veneris*) and *Christella dentata* are ferns that can function as hyperaccumulators of As and be used to effectively remove As from the soil [230]. Ye et al. (2011) found that *P. vittata* removed 3.5–11.4% of the total As in contaminated soil, and the content of As in the straw and grains was significantly reduced, and decreased phosphate-extractable As and soil pore water As by 11–38% and 18–77%, respectively, and lowed As concentrations in straw and grain, being 17–82% and 22–58% of those in the control, respectively [231]. However, the hyperaccumulators used in this method could have problems, such as small biomass, high cost for planting, the interruption of rotations that could limit agricultural production, and the occupation of farmland resources [232]. Therefore, screening or creating fast-growing and high-biomass plants that can become enriched in As could promote the popularization and application of phytoremediation approaches.

##### Seed Priming

Seed priming is a pre-exposure of seeds in the cellular state to an eliciting factor known to make the plants “prepared” for future harmful stress, thus resulting in greater survival under adverse environmental conditions [233]. It has been shown that priming techniques (e.g., external application of natural or synthetic compounds in plants) can enhance the tolerance of crops to environmental abiotic and biotic stress [234]. Seed priming enhances the resistance of rice to As. The technique involves the hydration of seeds with a sufficient amount of solvent to stimulate their metabolism before they actually germinate, and different types of organic or inorganic chemicals are often added during this process [235]. Accumulated As reduced germination, while the supplementation of selenium increased germination by 9%, indicating an apparent reduction in As accumulation [236]. Supplementation with zinc cation (Zn^2+^) by seed priming can effectively regulate redox homeostasis and reduce the absorption of As by rice seedlings due to its ability to limit the production of ROS. Additionally, the efficacy of Zn^2+^ seed priming on As-induced responses in developing rice seedlings is significantly influenced by varietal differences [237]. Although this technique is simple, rapid and easily popularized, its effectiveness in alleviating As stress in rice merits further exploration.

### 6.2. Biotechnological Pathways

**Figure 3:**
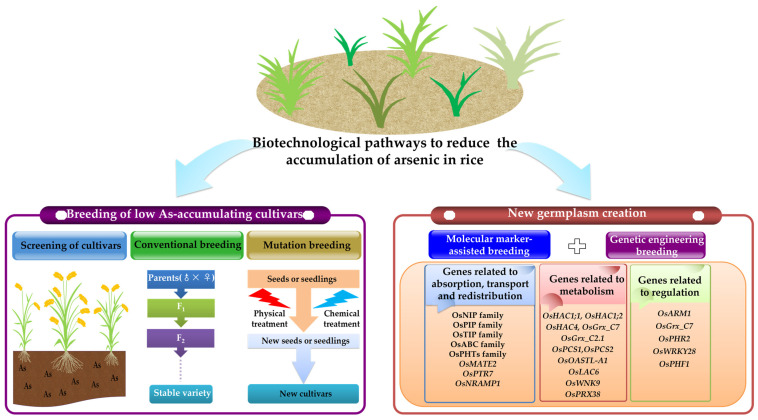
Biotechnological pathways to reduce the accumulation of arsenic (As) in rice.

Another important way to reduce the accumulation of As in rice is to cultivate varieties that contain low amounts of As. Such varieties can be produced by screening, conventional hybridization or mutagenesis, and the creation of new types of germplasm by genetic manipulation (Figure 3).

The production of varieties of rice that accumulate only low levels of As primarily includes two aspects: breeding and the creation of new germplasm. The breeding of new varieties entails ones that have been conventionally or treated with mutagens. The creation of new germplasm is primarily conducted to optimize the patterns of expression of the absorption, transport, and redistribution of As; its metabolism; and potential regulatory genes. In addition, favorable allelic variations can be explored, which would then enable the use of molecular-marker-assisted breeding, genetic engineering and other approaches to cultivate new rice materials that accumulate low levels of As.

#### 6.2.1. Breeding of Rice Varieties That Accumulate Low Levels of As

Owing to the differences in genetic characteristics of different rice varieties, the accumulation of As in grains can differ significantly. For example, the content of As in the grains of landraces with red bran is higher than that of landraces with brown bran [238]. The content of As in the grains of hybrid varieties is generally higher than that of conventional varieties [239]. In addition, the contents of As accumulated by varieties of *japonica* rice are relatively lower than those of the *indica* varieties [240]. Among the 76 rice varieties grown in Bangladesh, the concentration of As in the grains varied 4- to 5-fold [238]. Therefore, screening and cultivating rice varieties that accumulate low levels of As can effectively reduce the content of As in rice. Currently, the common methods of breeding include conventional, mutation, molecular-marker-assisted and genetic engineering among others [241]. However, there have been few studies on the breeding of rice varieties that accumulate low contents of As. The breeding of low cadmium rice provides a framework for breeding rice plants that accumulate low levels of As [242]. First, we propose to introduce the alleles for the low accumulation of As into excellent backbone parents through cross-breeding or to aggregate the alleles that control yield and resistance into varieties that accumulate low levels of As to obtain varieties of rice with high yields that are highly resistant to As. Secondly, Lim et al. (2020) used gamma rays to induce the production of a type 1 rice mutant *ATT1* that was tolerant to As, which was more effective at retaining As in vacuoles in the roots [243]. This laid a material foundation to cultivate new rice varieties that accumulate low levels of As by mutagenesis breeding. The combination of multiple methods can accelerate the efficiency and rate of success of breeding.

#### 6.2.2. Use of Genetic Engineering to Create New Rice Germplasm That Accumulates Low Levels of As

More than 40 genes involved in the absorption, transport, redistribution, metabolism and regulation of As in rice have been identified up to 2023. The genetic manipulation of these key genes by transgenic methods can result in the development of new rice germplasm that has low contents of As.

##### Genes Related to the Absorption, Transport, and Redistribution of As

The mutation of the aquaporin gene had a negative effect on the absorption of As(III) by the roots and its xylem loading. Compared with the WT, there was a significant reduction in the concentrations of As(III) in the roots and shoots of *oslsi1* mutant, and there was no significant difference in the concentration of As(III) in the grains, while the concentrations of As(III) in the shoots and grains of the *oslsi2* mutant were reduced by 13–19% and 51–63%, respectively [45]. Under anaerobic conditions, there was a decrease in the pH of root cells of rice due to the inhibition of alcohol dehydrogenase (ADH) in the *las3* mutant, which also inhibited the levels of expression of *OsLsi1* and *OsLsi2*, and reduced the accumulation of As(III) in the shoots and grains [56]. The knockout of *OsNIP3;2* significantly reduced the concentration of As in the rice roots but had no significant effect on the accumulation of As in the grains. The concentration of As in the roots of *osnip3;2* mutants that had been exposed to 5 µM and 20 µM concentrations of As(III) was 16–22% and 17–22% lower than that of the WT, respectively [36]. The overexpression of *OsPIP2;4*, *OsPIP2;6* or *OsPIP2;7* in *Arabidopsis thaliana* significantly enhanced the tolerance of transgenic plants to As [46].

Mutations in the genes that encode Pi transporters have led to a significant reduction in the accumulation of As in rice. Compared with the WT, less total As accumulated in the shoots of *ospt1* mutant [66]. In addition, the content of As in the roots of *ospt4* mutant decreased by 17–30% [244], while the absorption of As(V) by the roots of *ospt8* mutant decreased by 33–57% [68].

An increase in the root efflux of As(III) can reduce the accumulation of As in grains. The overexpression of *OsNIP1;1* and *OsNIP3;3* resulted in significantly lower concentrations of As in the brown rice, husk, leaves and stems of transgenic plants compared with the WT [34]. Overexpression of *OsNIP1;1* or *OsNIP3;3* provides a route for As(III) to leak out of the stele, thus restricting As(III) loading into the xylem, and therefore decreasing root-to-shoot translocation of As(III) and shoot As concentration markedly [34]. AtPIP2;2 promotes the efflux of As(III) and improves the tolerance of *A. thaliana* to As(III) stress [245]. Whether OsPIP2;2 also has a similar efflux function in rice merits further study.

The genes related to the absorption, transport and redistribution of As in rice that have been identified so far are not very specific. The modification of these genes may have adverse effects on the growth of rice while reducing the accumulation of As, which requires comprehensive consideration. Subsequent research can further explore the specific genes for the absorption and transport of As in rice.

##### Genes Related to Metabolism

The patterns of expression of the reduction, chelation and other genes related to the metabolism of As in rice were optimized to limit the accumulation of As by improving the physiological and biochemical metabolism of rice.

The overexpression of *OsHAC1;1* and *OsHAC1;2* can reduce the accumulation of As and improve the tolerance to it [76]. The efflux of As(V) to the growth medium increased in the overexpression lines that were planted in paddy fields contaminated with As(V), which resulted in a concentration of As in the grain that was approximately 20% lower than that of the WT [76]. The concentration of As in the roots and shoots of *OsHAC4* that overexpressed the transgenic rice decreased by 39.2–42.1% and 38.2–40%, respectively, while the loss of function of *OsHAC4* resulted in a decrease in the efflux of As(III) and an increase in the concentration of As in the shoots [78]. The overexpression of *OsGrx_C7* and *OsGrx_C2.1* in *A. thaliana* increased the levels of expression of *AtNIP1;1*, *AtNIP2;1* and *AtNIP7;1*; decreased the accumulation of As in the seeds and shoots, and enhanced the tolerance to As [48,58]. The *PvACR3* gene from *P. vittata* was heterologously expressed in rice, and the content of inorganic As in the grains of transgenic rice decreased by 26–46% [246]. The *ScACR3* gene of yeast was transferred into rice, and it significantly reduced the accumulation of As in the rice straw and grains [247].

Glutathione S-transferases (GSTs) catalyze the combination of As(III) and GSH to form As(III)-GSH complexes with GSH as a substrate [40]. *OsGSTU5* was involved in the chelation of As(III) with GSH and then isolated the chelate into the root vacuoles [59]. The PC synthase genes *OsPCS1* and *OsPCS2* catalyze the biosynthesis of PC [51]. The overexpression of *OsPCS1* resulted in a 75% decrease in the content of As in the grain [50]. The overexpression of the PC synthase gene *CdPCS1* from coontail (*Ceratophyllum demersum*) in rice resulted in a decrease in the content of As in the husks and grains [248]. The mutation of *OsCLT1* resulted in a decrease in the contents of GSH and PCs in the cytoplasm [47]. Under As(V) treatment, the *osclt1* mutant accumulated 50% less As in its roots than the WT, while the accumulation of As in the shoots increased significantly [47]. The exposure of rice plants with the *OsOASTL-A1* mutant to As(V) resulted in a decrease in the accumulation of cysteine, GSH, PCs and As in the roots, while the accumulation of As in the shoots was higher than that of the WT by 36–68% [80]. In addition, OsABCC1 mediates the transport of As(III)-PC complex from the cytoplasm to the vacuole for storage, thereby reducing the accumulation of As in the rice grains. OsABCC7 promotes the translocation of As(III) from the roots to shoots through the efflux of As(III)-PC2 and As(III)-GS3 [44]. The simultaneous overexpression of the genes for GST and PC synthase and the *OsABCCs* in rice can promote the chelation of As and its compartmentation in the vacuole to the greatest extent, which provides a good strategy to create new rice germplasm that accumulates low levels of As.

The overexpression of *OsLAC6* significantly increased the activity of laccase in the leaves of transgenic rice lines, which reduced the content of lignin in the roots and the plant’s tolerance to As(III) [55]. Under As(III) stress, the content of proline in *A. thaliana* lines that overexpressed *OsWNK9* was significantly higher than that of the WT, and the biomass increased by 52–58% [54]. Overexpression of the rice type III peroxidase gene *OsPRX38* in *A. thaliana* activates the antioxidant signal network, promotes the biosynthesis of lignin, increases the degree of root lignification, hinders the absorption of As through lignification of the apoplast, and reduces the accumulation of As [52]. Subsequent studies can verify whether these genes can also play a role in rice and be applied to genetic engineering to create rice that accumulates low levels of As.

The screening and functional verification of the genes related to metabolism can be conducted together with the development of efficient physiological regulators. While improving the metabolic pathway of rice to alleviate As toxicity, new physiological regulators can be developed for the core regulatory factors once their mechanism of action is clarified to improve the amount of control and feasibility of application in actual production.

##### Genes Related to Regulation

The TFs that regulate the absorption, transport, and redistribution of As in rice and the genes related to metabolism at the transcriptional level can be used for genetic engineering to create rice that accumulates low levels of As. When treated with 30 μM of As, which induced stress, the OsPT2 and OsPT8 transporters in the *osphf1* knockout mutant were retained in the endoplasmic reticulum, and the tolerance to As increased [249]. The knockout of *OsARM1* resulted in an up-regulation of the expression of As(III) transporter genes, such as *OsLsi1*, *OsLsi2* and *OsLsi6*, increased the content of As content in the shoots and increased the resistance to As(III), while the overexpression lines manifested the opposite results [57]. The TF OsWRKY28 regulates the accumulation of P and As(V) in rice. The knockout of this gene resulted in changes in the levels of expression of 13 Pi transporter genes, and the concentration of As that accumulated in the rice shoots was reduced by 16–31% [70]. In addition, the microRNAs involved in post-transcriptional regulation can also be used as gene resources to breed rice that accumulates low levels of As.

There are transcriptional, post-transcriptional and post-translational regulatory mechanisms in rice, and few regulatory genes related to the stress responses to As have been identified. Thus, knowledge of the complete upstream and downstream molecular signal transduction pathways remains unclear. The possibility that there may be multiple target genes of the upstream key regulatory factors increases the probability of optimizing them to obtain their target traits. Therefore, further mining of the TFs related to As and their interacting proteins, and microRNAs among others should help facilitate the improvement of the efficiency of creating new germplasm of rice that accumulates low levels of As.

## 7. Conclusions and Prospects

**Figure 4:**
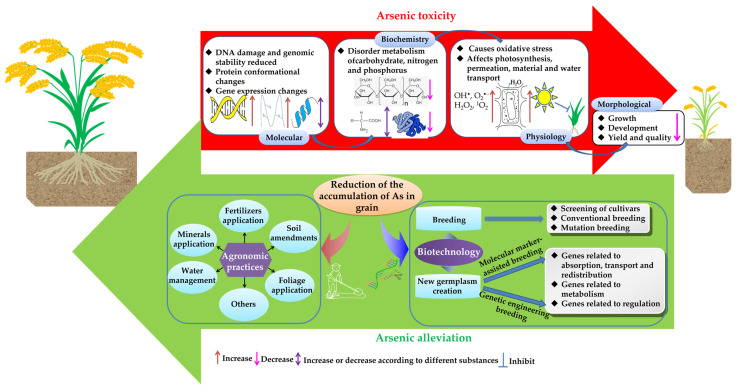
The effects of arsenic toxicity on rice and the strategies to reduce arsenic accumulation in grain.

Arsenic stress leads to the inhibition of rice growth and development, disorders of the physiological and biochemical metabolic systems, and a decrease in the yield and quality of rice. Revealing the molecular mechanism of the absorption, transport, redistribution and metabolism of As in rice and identifying the genes (such as the genes in Table 1, Table 2 and Table 3) and their products that are related to As tolerance will help to reduce the accumulation of As in rice by agronomic measures (such as application of minerals, soil amendments and fertilizers, foliar spray, water management, etc.). Another option is to use genetic engineering (such as traditional and modern breeding, etc.) to create such lines of rice (Figure 4). Since the rice varieties created by transgenic technology may be limited to promotion globally now, what we are doing is studying the functions of these genes and then cloning them to obtain key core gene resources, to find their favorable alleles in different varieties, and create new rice varieties through gene polymerization molecular breeding. At present, many new crop varieties constructed by gene editing have been liberalized in many regions, but they are not much recommended to promote globally, let alone the transgenic rice varieties through transgenic technology.

However, there are still many problems that merit further study and discussion: (1) Research on the use of minerals as supplements to reduce the accumulation of As in rice has been performed the most on iron, P, S, Si, Se and Mn, while only Olyaie et al. (2012) [250] and Syu et al. (2020) [218] found that the use of calcium peroxide (CaO_2_) can help to reduce the toxicity and mobility of As in soil, remove As from water [250], or effectively reduce the accumulation of As in rice [218]. In the future, the effects of applying CaO_2_ by flooding application on the dynamics of As in the soil and its absorption by rice and the mechanism used can be studied in more detail. (2) Engineered nanomaterials (ENMs) are nanomaterials conceived, designed, and intentionally produced by humans from numerous types of nanomaterials with a size of 100 nm or less [251,252]. Although the application of ENMs helps to reduce the accumulation of As in rice, it also has adverse effects on soil microorganisms and plants. The consumption of grains, fruits and vegetables that have been harvested from the soil that contains ENMs can indirectly lead to human exposure to environmental nano-pollutants that pose potential risks to health. In the future, it is necessary to clarify the effects of ENMs on the soil (microorganism)–rice–human body, conduct extensive and in-depth ecotoxicological research, and develop new engineering nanomaterials for plants. (3) The management of water can reduce the content of As, but it will also lead to a reduction in the yield of rice. The ability to attain the best balance of stable yield, water conservation and a reduction in As through precise water management is a difficulty that needs to be overcome in the future water management of paddy fields. (4) The use of microbial biofertilizers to reduce the effectiveness and toxicity of As is a promising research direction. It is also necessary to strengthen the interaction between microorganisms in microbial biofertilizers and the natural flora in the soil, as well as the field application of microbial biofertilizers. (5) The use of phytoremediation can result in problems, such as the small biomass of hyperaccumulators, occupation of farmland resources, and difficulty in implementing rice rotations. A feasible way to improve the effect of phytoremediation is the use of genetic engineering to develop hyperaccumulators with a large biomass that is suitable for rice rotation. (6) Some researchers have proposed a method to reduce the accumulation of As in grains by altering the distribution of As in rice and retaining it in the vegetative organs, such as the roots or stems and leaves. However, this method could lead As to enter the food chain again through feed. Therefore, a comprehensive risk assessment needs to be conducted before this approach is implemented [63]. (7) Some gene resources that have been identified to be involved in the accumulation of As in rice can be applied to genetic engineering, but the pattern of expression should be optimized and considered comprehensively from the overall level. Most genes are not specific to As and have some other functions, are transport-related and are involved in various cellular functions, so changes in expressions of these genes may affect the absorption of other substances, especially beneficial elements. For example, the gene knockouts of some Pi and silicon transporters (such as *OsLsi1*, *OsPT1*, etc.) can reduce the absorption of As in rice [63], but they can also lead to a reduction in the absorption of nutrients, such as phosphate ion and orthosilisic acid, which results in stunted rice growth and reduced yields. Therefore, achieving the win–win benefit of As reduction and stable yield by selecting appropriate transporter genes and optimizing their patterns of expression will be the basis of successfully cultivating a new germplasm. It should be considered modifying these kinds of genes via gene editing for the desired function alone or to inactivate the undesired function of an encoding part of a specific domain or motif of its protein. Meanwhile, As and its metabolites can interact with zinc finger motifs and RING finger domains, disrupting the functions of proteins and may lead to multiple adverse effects, so it is necessary to focus on specific motifs or domains that bind to As, and use biotechnology to carry out site-specific mutations on these specific motifs or domains to achieve the purpose of avoiding the competitive or targeted binding of As to these targets, but without affecting the absorption of other components (especially nutrient elements) in the future. In addition, the excavation of homologous genes of key genes in As stress response in other species, such as *A. thaliana* and maize (*Zea mays*), in rice will facilitate an accelerated expansion of gene resources for tolerance to As. Simultaneously, the breeding of low As rice should be closely combined with modern agronomic practices to remediate each other’s defects and achieve the purpose of ‘curing the symptoms and curing the root cause’.

## Figures and Tables

**Table 1:** Genes involved in the absorption, transport and redistribution of As(III) in rice.

Function	Gene Name	Locus ID	Mechanism	Refs.
Absorption and transport	*OsABCC1*	LOC_Os04g52900	Promotes the transport of As(III) to the vacuole for sequestration	[42]
	*OsABCC2*	LOC_Os01g67580	Transports As-PC complexes	[43]
	*OsABCC7*	LOC_Os04g49900	Promotes the loading of As(III) inside the xylem	[44]
	*OsLsi2*	LOC_Os03g01700	Effluxes As(III) from the root to xylem and phloem	[45]
	*OsNIP1;1*	LOC_Os02g13870	Provides a route for As(III) to leak out of the stele, thus, restricting As(III) loading into the xylem	[34,45]
	*OsNIP2;1 (OsLsi1)*	LOC_Os02g51110	Transports As(III) into the root	[45]
	*OsNIP2;2 (OsLsi6)*	LOC_Os06g12310		
	*OsNIP3;1*	LOC_Os10g36924	Slightly absorbs As(III) in rice	[45]
	*OsNIP3;2*	LOC_Os08g05590	Promotes the absorption of As(III) by lateral roots	[36]
	*OsNIP3;3*	LOC_Os08g05600	Provides a route for As(III) to leak out of the stele, thus, restricting As(III) loading into the xylem	[34]
	*OsNRAMP1*	LOC_Os07g15460	Promotes xylem loading for the root-to-shoot mobilization	[37]
	*OsPIP2;4*	LOC_Os07g26630	Influxes and effluxes of As(III) in the roots	[46]
	*OsPIP2;6*	LOC_Os04g16450		
	*OsPIP2;7*	LOC_Os09g36930		
Metabolism	*OsCLT1*	LOC_Os01g72570	Maintains glutathione homeostasis and a higher accumulation of As(III) in the roots	[47]
	*OsGrx_C2.1*	LOC_Os02g40500	Involved in redox regulation and protection under oxidative stress/Reduces the accumulation of intracellular As	[48]
	*OsGrx_C2.2*	LOC_Os04g42930	Maintains cellular redox homeostasis	[49]
	*OsPCS1*	LOC_Os05g34290	Promotes PC synthesis	[50]
	*OsPCS2*	LOC_Os06g01260		[51]
	*OsPRX38*	LOC_Os03g13210	Activates the signaling network of different antioxidant systems under As stress conditions and enhances tolerance by reducing the accumulation of As due to apoplastic lignification	[52]
	*OsSultr1;1*	LOC_Os03g09970	Enhances the activity of antioxidants	[53]
	*OsWNK9*	LOC_Os12g06490	Increases antioxidant capacity, proline and reduces the levels of hydrogen peroxide	[54]
	*OsLAC6*	LOC_Os01g62600	Increases the activity of laccase in the leaves, decreases the lignin content in the roots, and negatively regulates tolerance to As(III)	[55]
Regulation	*OsADH2*	LOC_Os11g10510	Regulates silicate transporters to influence the contents of As(III) in the aerial tissues of rice	[56]
	*OsARM1*	LOC_Os05g37060	Regulates the absorption and root-to-shoot translocation of As(III)	[57]
	*OsGrx_C7*	LOC_Os01g27140	Regulates the levels of expression of the aquaporins, which reduce the translocation of As(III) to the roots	[48,58]
	*OsGSTU5*	LOC_Os09g20220	Promotes the chelation of As with GSH and sequesters it into the root vacuole using the OsABCC1 transporter and thus, limits the upward translocation of As toward the shoot and maintains the ROS homeostasis, physiological and biochemical activities.	[59]

**Table 2:** Genes involved in the absorption, transport and redistribution of As(V) in rice.

Function	Gene Name	Gene ID	Mechanism	Refs.
Absorption and transport	*OsPT1*	LOC_Os03g05620	Promotes the absorption and translocation of As(V) from the roots to shoots	[66]
	*OsPT4*	LOC_Os09g37200		[67]
	*OsPT8*	LOC_Os10g30790	Promotes the absorption of As(V)	[68,69]
Metabolism	*OsHAC1;1*	LOC_Os02g01220	Promotes the reduction of As(V) to As(III)	[76,77]
	*OsHAC1;2*	LOC_Os04g17660		[76]
	*OsHAC4*	LOC_Os02g06290		[78]
	*OsACR2.1*	LOC_Os10g39860	Can act as arsenate reductases	[79]
	*OsACR2.2*	LOC_Os03g01770		
	*OsOASTL-A1*	LOC_Os03g53650	Plays an important role in the biosynthesis of non-protein thiols in the roots to detoxify As	[80]
Regulation	*OsPHF1*	LOC_Os07g09000	Regulates OsPT8 for the absorption and transport of As(V)	[69]
	*OsPHR2*	LOC_Os07g25710	Regulates Pi transporters to affect the transport of As(V) to the roots and xylem	[69]
	*OsNLA1*	LOC_Os07g47590	Primarily regulates As(V) absorption and tolerance by regulating the amount of Pi transporters	[81]
	*OsWRKY28*	LOC_Os06g44010	Regulates the accumulation of As(V) in the shoots	[70]
	*OsCLT1*	LOC_Os01g72570	Maintains GSH homeostasis by mediating the export of γ-glutamylcysteine and GSH from plastids to the cytoplasm, which, in turn, affects As detoxification in rice	[47]
	*OsMATE2*	LOC_Os05g48040	Modulates the accumulation of As in rice grains	[82]

**Table 3:** Genes involved in the absorption, transport and redistribution of DMA or MMA in rice.

Function	Gene Name	Locus ID	Mechanism	Ref.
Absorption and transport	*OsNIP2;1 (OsLsi1)*	LOC_Os02g51110	Transports of DMA and MMA into the root and upward	[90]
	*OsPTR7*	LOC_Os01g04950	Involves in the long-distance transport of DMA	[95]
Redistribution	*OsPTR7*	LOC_Os01g04950	Involves in the accumulation of DMA in the rice grains	[95]

## Data Availability

Not applicable.

## Abbreviations

ABC	ATP-binding cassette
ABCC	C-TYPE ABC subfamily transporter
ABCG	ABC transporter G family member
ACA	P2B-type autoinhibited Ca^2+^-ATPase
ACR	arsenate reductase
ADH	alcohol dehydrogenase
ALA	P4-type aminophospholipid ATPase
ARM	ARSENITE-RESPONSIVE MYB
As	arsenic
As(III)	arsenite
As(III)-PC	As(III)-phytochelatin complex
As(V)	arsenate
AUX	auxin
CAT	catalase
CLT	CRT-like transporter
DMA	dimethylarsenic acid
DNA	deoxyribonucleic acid
Grx	glutaredoxin
Grx_C7	glutaredoxin_C7
GSH	glutathione
GSTU	glutathione-S-transferase
H_2_O_2_	hydrogen peroxide
HAC	high arsenic content
HMA	heavy metal ATPase
IAA	Indole-3-Acetic Acid
Lsi	low silicon
MATE	multidrug and toxic compound extrusion
MIP	membrane intrinsic proteins
MMA	monomethylarsonic acid
MYB	v-myb avian myeloblastosis viral oncogene homolog
NIP	nodulin26-like intrinsic protein
NLA	nitrogen limitation adaptation
NO	nitric oxide
NRAMP	natural resistance macrophage protein transporter
OASTL-A1	O-acetylserine(thiol) lyase 1
PC	phytochelatin
PCS	phytochelatin synthase
PHF	phosphate transporter traffic facilitator
PHO	phosphate
PHR	phosphate starvation response
PIP	plasma membrane intrinsic protein
PRX	peroxidase
PTR	putative peptide transporter
RNS	reactive nitrogen species
ROS	reactive oxygen species
SA	salicylic acid
SH	sulfhydryl
Si	silicon
SIP	small basic intrinsic proteins
SLs	strigolactones
SOD	superoxide dismutase
Sultr(SULTR)	sulphate transporter
TIP	tonoplast intrinsic protein
TMA	trimethylarsine
WNK	with no lysine
WT	wild type
XIP	uncategorized intrinsic proteins
